# The design of retroviral vectors used in the CAR‐T products, risk management, and future perspective

**DOI:** 10.1002/mco2.70067

**Published:** 2025-01-24

**Authors:** Huifang Yin, Xuejing Wei

**Affiliations:** ^1^ Office of Pharmaceutical Science Yangtze River Delta Center for Drug Evaluation and Inspection National Medical Products Administration Shanghai China

**Keywords:** CAR‐T therapy, replication‐competent retroviruses (RCR), retroviral vectors, risk management, secondary malignancies, SIN vectors

## Abstract

Chimeric antigen receptor T‐cell (CAR‐T) therapy is a revolutionary approach in cancer treatment. More than 10 CAR‐T products have already approved on market worldly wide, and they use either gamma retroviral vectors or lentiviral vectors to deliver the CAR gene. Both vectors have the ability to effectively and persistently integrate the CAR gene into T cells. Despite the advancements in CAR‐T therapy, the potential risks associated with the vectors, particularly the risks of the secondary malignancies, still remain as a concern. This article compares the characteristics of gamma retroviral and lentiviral vectors, discusses the development of vector packaging systems, and examines the design of self‐inactivating (SIN) vectors. It also addresses the risks of secondary malignancies that might possibly be associated with the retroviral vectors, and the strategies to decrease the risks and increase the safer clinical use of the vectors. This article also discusses the current regulatory landscape and management approaches aiming to mitigate these risks through stringent safety measures and ongoing monitoring. Future perspectives focus on improving the safety profiles of the vectors and broadening their scope of use. The article provides a thorough overview of the most recent research discoveries and regulatory updates in the field of CAR‐T therapy, highlighting the significance of a balanced strategy that strikes a balance between innovation and patient safety in the development and implementation of CAR‐T therapy.

## Graphical abstract

This article compares gamma retroviral and lentiviral vectors, discussing packaging systems and SIN vector design. It also covers risks of secondary malignancies linked to retroviral vectors and strategies to minimize them. The article explores the regulatory framework, safety monitoring, and future safety improvements for CAR‐T therapy, emphasizing a balance between innovation and patient safety.

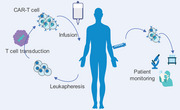

## 1 INTRODUCTION

Adoptive cell therapy with chimeric antigen receptor (CAR) immunotherapy has made remarkable progress for hematological malignancies due to the rapid and durable clinical responses.^1^ CARs can target T cells to the specific tumor‐associated antigen on the cell surface and induce an anticancer immune response, which can efficiently eliminate the tumor cells. As of April 2024, there are 11 CAR‐T products approved globally, of which six of them were first approved in the United States, namely, tisagenlecleucel,^2^, ^3^, ^4^, ^5^ axicabtagene ciloleucel,^6^, ^7^, ^8^, ^9^ brexucabtagene autoleucel,^10^, ^11^ lisocabtagene maraleucel,^12^, ^13^, ^14^ idecabtagene vicleucel,^15^, ^16^, ^17^ and ciltacabtagene autoleucel^18^, ^19^, ^20^; five of them were approved in China, namely, axicabtagene ciloleucel, relmacabtagene autoleucel,^21^, ^22^ equecabtagene autoleucel,^23^, ^24^, ^25^ inaticabtagene autoleucel,^26^ and zevorcabtagene autoleucel.^27^, ^28^ The information of the approved CAR‐T products and the used retroviral vectors is summarized in Table 1.

**TABLE 1:** The information of the approved chimeric antigen receptor T‐cell (CAR‐T) products and their retroviral vectors.

Tradename	Kymriah	Yescarta	Tecartus	Breyanzi	Abecma	Carvykti	Yikaida	Beinuoda	Fukesu	Yuanruida	Saikaize
**Proper name**	Tisagenlecleucel	Axicabtagene ciloleucel	Brexucabtagene autoleucel	Lisocabtagene maraleucel	Idecabtagene vicleucel	Ciltacabtagene autoleucel	Axicabtagene ciloleucel	Relmacabtagene autoleucel	Equecabtagene autoleucel	Inaticabtagene autoleucel	Zevorcabtagene autoleucel
**Manufacturer**	Novartis Pharmaceuticals Corporation	Kite Pharma Inc.	Kite Pharma, Inc.	Juno Therapeutics, Inc.	Celgene Corporation	Janssen Biotech, Inc.	FOSUNKite	JW Therapeutics	IASO Bio	Juventas Cell Therapy Ltd	Carsgen Therapeutics
**First approval date**	August 30, 2017	October 18, 2017	July 24, 2020	February 5, 2021	March 26, 2021	February 28, 2022	June 22, 2021	September 1, 2021	June 30, 2023	November 8, 2023	March 1, 2024
**First approval country**	USA	USA	USA	USA	USA	USA	China	China	China	China	China
**Indication**	R/R B‐ALL (2017) LBCL after ≥2 lines of therapy (2018) FL after ≥2 lines of therapy (2022)	Relapsed LBCL after ≥2 lines of therapy (2017) Relapsed FL after ≥2 lines of therapy (2021) LBCL refractory to first‐line therapy (2022)	R/R MCL (2020) R/R B‐ALL (2021)	Relapsed LBCL after ≥2 lines of therapy (2021) LBCL refractory to first‐line or relapsing at <12 months of first line therapy or relapsing on first‐line therapy and not eligible for HSCT (2022) Adult patients with R/R CLL or SLL who have received at least two prior lines of therapy (2024)	Fifth line RRMM (2021)	Fifth line RRMM (2022) Patients with RRMM who have received at least one prior line of therapy (2024)	R/R LBCL (2021) LBCL (2023)	R/R LBCL (2021) R/R FL (2022)	Third line RRMM (2023)	R/R B‐ALL (2023)	Third line RRMM (2024)
**Target antigen**	CD19	CD19	CD19	CD19	BCMA	BCMA	CD19	CD19	BCMA	CD19	BCMA
**Antigen‐binding domain origin**	Mouse‐derived antibody					Human‐derived antibody, two linked camelid heavy‐chain‐only variable domains	Mouse‐derived antibody		Fully human‐derived antibody	Unknown	Fully human‐derived antibody
**Costimulatory domain**	4‐1BB	CD28		4‐1BB			CD28	4‐1BB			

Tradename	Kymriah	Yescarta	Tecartus	Breyanzi	Abecma	Carvykti	Yikaida	Beinuoda	Fukesu	Yuanruida	Saikaize
**Proper name**	Tisagenlecleucel	Axicabtagene ciloleucel	Brexucabtagene autoleucel	Lisocabtagene maraleucel	Idecabtagene Vicleucel	Ciltacabtagene Autoleucel	Axicabtagene ciloleucel	Relmacabtagene autoleucel	Equecabtagene autoleucel	Inaticabtagene autoleucel	Zevorcabtagene autoleucel
**Vector type**	HIV‐1‐derived replication‐incompetent, third‐generation SIN lentiviral vector pseudotyped with VSV‐G	MSCV‐based vector pseudotyped with the GaLV envelope, non‐self‐inactivating gamma retroviral vector		HIV‐1‐derived replication‐incompetent, third‐generation SIN lentiviral vector pseudotyped with VSV‐G			MSCV‐based vector pseudotyped with GaLV envelope, non‐self‐inactivating gamma retroviral vector	HIV‐1‐derived replication‐incompetent, third‐generation SIN lentiviral vector pseudotyped with VSV‐G	HIV‐1‐derived replication‐incompetent, third‐generation SIN lentiviral vector pseudotyped with VSV‐G (speculated)		
**Vector production**	Transient transfection of HEK293T cells	Stable transfection of packaging cell clone PG13‐CD19‐H3		Transient transfection of HEK293T cells			Stable transfection of packaging cell clone PG13‐CD19‐H3	Transient transfection of HEK293T cells (speculated)			
**Promoter**	EF‐1α promoter	MSCV promoter		Unknown	Murine leukemia virus–derived MND promoter	EF‐1α promoter	MSCV promoter	Unknown			

*Note*: The information is obtained from United States Food and Drug Administration website, European Medicines Agency website, and the cited references.

Abbreviations: B‐ALL, B‐cell acute lymphoblastic leukemia; CLL, chronic lymphocytic leukemia; EF‐1α promoter, human elongation factor 1α; FL, follicular lymphoma; GaLV, the gibbon ape leukemia virus; HSCT, hematopoietic stem cell transplantation; LBCL, large B‐cell lymphoma; MCL, mantle cell lymphoma; MSCV, murine stem cell virus; R/R, relapsed and/or refractory; RRMM, R/R multiple myeloma; SLL, small lymphocytic lymphoma. SIN, self‐inactivating; VSV‐G, vesicular stomatitis virus glycoprotein.

Most of the approved CAR‐T products have similar CAR structures, a single‐chain variable fragment (except ciltacabtagene autoleucel, which has two linked camelid heavy‐chain‐only variable domains) that recognizes and binds to the target antigen, a hinge and transmembrane region, a costimulatory domain, and a T‐cell activation domain.^29^ Moreover, they all use retroviral vectors for CAR gene delivery, exploiting their stable genome integration and higher efficiency than DNA transfection.^30^ While retroviral vectors offer durable CAR‐T efficacy, they pose risks like replication‐competent retroviruses (RCRs) and insertional mutagenesis, potentially activating oncogenes or causing secondary malignancies.^31^, ^32^, ^33^, ^34^ These risks are drawing increasing attention with the rapid development of CAR‐T therapy.

We begin this review by comparing the gamma retroviral vectors and lentiviral vectors used in the approved CAR‐T products. Next, this paper goes over the evolution of vector packaging systems and talks about the SIN vector design. In the next part, we discuss the most recent regulatory management toward the secondary malignancies, the possible causation, and the strategies to decrease the risks. Finally, future perspectives toward a safer use of retroviral vectors and advices toward the patient monitor are raised.

## 2 THE RETROVIRAL VECTORS USED IN THE CAR‐T PRODUCTS

As shown in Table 1, all the approved CAR‐T products use gamma retroviral vectors or lentiviral vectors for the delivery of the CAR genes. Both axicabtagene ciloleucel (Yescarta, Kite Pharma Inc.; FOSUNKite) and brexucabtagene autoleucel (Tecartus, Kite Pharma, Inc.) use gamma retroviral vector, a murine stem cell virus‐based vector pseudotyped with the gibbon ape leukemia virus (GaLV) envelope, while the other products use lentiviral vector, a HIV‐1‐derived replication‐incompetent vector pseudotyped with the vesicular stomatitis virus glycoprotein (VSV‐G). Gamma retroviruses and lentiviruses belong to different genera of *Retroviridae* family, both containing two copies of positive‐sense single‐stranded RNA (ssRNA) genome that is reverse‐transcribed into DNA in the transduced cell by a virally encoded enzyme called reverse transcriptase.^35^, ^36^, ^37^ Vectors derived from gamma retroviruses and lentiviruses are widely used in the cell and gene therapy because of their natural ability to stably integrate their genes into the host cell genome.^35^, ^36^, ^38^ Gamma retroviruses can only gain access to the host genome during mitosis, when the nuclear membrane is breakdown.^35^, ^36^, ^37^ On the contrary, lentiviruses can translocate across the nuclear pore of an intact nuclear envelope, allowing transduction of both proliferating and non‐proliferating cells.^35^, ^36^, ^37^ Cells may retain a greater functional potential, and a lower oncogenic potential when they are transduced in their non‐dividing state.^35^ The integration process is not random, with gamma retroviruses preferring insertion near transcriptional start sites and regulatory regions, while lentiviruses insert within the transcriptional units.^35^, ^36^, ^39^ Therefore, there is an increased chance for gamma retroviral vectors to insert into a regulatory region that involved in cell division when transducing a dividing cell. The intrinsic integration properties enable the retroviral vectors to insert the gene of interest (GOI) into the genome, and meanwhile also bring some risks. The insertion may cause disruption or activation of the neighboring host genes, which may potentially lead to insertional mutagenesis and tumorigenesis. The infectious titers depend on numerous factors, including packaging cell lines, envelopes, and the production medium. As the production process is refined, both gamma retroviral vectors and lentiviral vectors can achieve a titer of over 10⁶ infectious particles per milliliter of cell culture.^40^ A comparison of the two vectors is provided in Table 2.

**TABLE 2:** The comparison of gamma retroviral vectors and lentiviral vectors.

	Gamma retroviral vectors	Lentiviral vectors
Transduction property	Dividing cells	Both dividing and non‐dividing cells
Integration pattern	Permanent integration	Permanent integration
Integration site	Prefer transcriptional start sites and regulatory gene regions	Prefer within the transcriptional units
Transfection efficiency	High to dividing cells	High
Clinical safety	Higher than lentiviral vectors	Relatively low

Notably, the gamma retroviral vectors used in axicabtagene ciloleucel (Yescarta; Yikaida) and brexucabtagene autoleucel (Tecartus) are non‐self‐inactivating vectors while other lentiviral vectors are self‐inactivating (SIN) vectors. This distinction arises from their production methods: the gamma retroviral vectors are produced using stably transduced PG13 cell lines, expressing the GALV envelope and the Moloney murine leukemia virus gag‐pol proteins, while the lentiviral vectors are produced by transient transfection of HEK293T cells. While SIN vectors can be produced through transient transfection, they may not be produced by stable packaging cell lines, because in retroviral vector transduced packaging cell lines the SIN long terminal repeat (LTR) cannot produce packageable genomic viral RNA.^38^ The trend is now to use a transient production system to produce the SIN vectors, which is flexible and efficient. On the other hand, it is also expensive and the quality varies from batch to batch, which may affect the reproducibility of the manufacturing process and the quality of the final cell products. The stable packaging cell lines allow scalable and cost‐effective viral vector production, but the construction of the cell lines is time consuming and difficult to produce the safer SIN vectors. To improve reproducibility and safety, efforts are ongoing to develop stable packaging cell lines capable of producing SIN vectors.^41^, ^42^, ^43^

## 3 DEVELOPMENTS OF THE VECTOR PACKAGING SYSTEMS

Retroviral vectors are constructed by inserting the GOI into the retroviral genome and take advantage of all necessary retroviral proteins for the infection. Retroviral vectors and in particular HIV‐derived lentiviral packaging systems have been extensively optimized to minimize RCR generation and adverse integrations. Strategies include (but not limited to) deleting unnecessary accessory genes for the vector production, splitting the essential genes into separate plasmids, minimizing the sequence overlapping between different plasmids, and designing SIN vector. The packaging systems have evolved through three generations, significantly enhancing clinical safety.

The first‐generation system was developed by Naldini et al.,^44^ containing three plasmids, a transfer plasmid harbors the GOI, a packaging plasmid containing the *gal*, *pol, rev, tat* genes, and the accessory genes (*vif*, *vpr*, *vpu*, and *nef*), and an envelope plasmid expressing the glycoprotein G from the VSV‐G.^45^, ^46^ The second generation system improved the safety of the system by rendering the lentivirus non‐pathogenic by eliminating the accessory genes (*vif*, *vpr*, *vpu*, and *nef*) in the packaging plasmid, and vector production was not affected with the loss of these genes.^45^, ^46^ The third‐generation system further refined safety by deleting the *tat* gene in the packaging plasmid, and splitting the *rev* gene into a separate plasmid, resulting in a four‐plasmid system.^45^, ^46^ The third‐generation transfer vector further deleted the U3 region in the LTR, which contains the viral promoter and enhancer. The deletion of U3 region diminishes the upregulation or disruption of neighboring genes at the insertion sites and minimizes the risks of vector mobilization and recombination.^45^ The *tat* gene, crucial for LTR‐driven viral RNA transcription, is also excised from the packaging vector as the RNA expression is no longer driven by the LTR.^46^ A comparison of the three‐generation lentiviral systems is summarized in Table 3.

**TABLE 3:** Comparison of the three‐generation lentiviral systems.

	First generation	Second generation	Third generation
Plasmids	3	3	4
Accessory genes	*vif, vpr, vpu, nef*	none	none
*tat* gene	On packaging plasmid	On packaging plasmid	absent
*rev* gene	On packaging plasmid	On packaging plasmid	On a separate plasmid
SIN vector	No	No	Yes

In the third‐generation system, genes encode proteins essential for lentiviral vector production. The *gag* gene encodes structural proteins such as the matrix, capsid, and nucleocapsid,^36^, ^47^ which are part of protein scaffolds that act as a protective layer for the viral RNA genome.^48^ The *pol* gene encodes enzymes crucial for virus replication and maturation, including protease, reverse transcriptase, and integrase.^47^ The protease cleaves HIV immature proteins into mature functional proteins; the reverse transcriptase reverse‐transcribes the viral genomic ssRNA into double‐stranded DNA (dsDNA); and the integrase incises the host genomic DNA to integrate the viral dsDNA.^49^ The *env* gene encodes for the envelope glycoprotein and transmembrane domains, which are responsible for the tropism by interacting with the specific cellular receptors, and mediating fusion and cell entry with the cell membrane.^36^, ^47^ VSV‐G, now a standard for lentiviral vector production, recognizes the low‐density lipoprotein (LDL) receptor, which is ubiquitously expressed on cell surfaces, enabling transduction across a wide cell range and broadening the tropism.^35^, ^50^ Additionally, VSV‐G pseudotyping allows efficient vector concentration by ultracentrifugation without compromising biological activity, leading to high‐titer lentiviral vector production.^36^, ^50^, ^51^ The *rev* gene mediates the export of the intact and singly spliced viral RNAs from the nucleus, thus permitting the expression of the *gag*, *pol*, and *env* gene products.^52^

Although the optimization of retroviral vectors greatly increased the safety of clinical use, the risks associated with the vectors still remain and require further attention. For example, a fourth‐generation lentiviral system was designed to further minimize the sequence homology between plasmids and eliminated the need for the *rev* plasmid by codon optimizing the *gal* and *pol* genes.^45^ Additionally, incorporating enhancer‐blocking insulators into retroviral vectors can hinder interactions between integrated genes and the target cell genome, further decreasing the chances of insertional oncogenesis.^53^, ^54^ As cell tropism is mediate by the virus envelope, the pseudotyped VSV‐G envelope is able to transduce a wide range of cells and have a broad tropism, but they cannot confer efficient transduction in quiescent cells such as hematopoietic stem cells, B cells, and T cells.^55^ Also, the VSV‐G lentiviral vectors can be inactivated by human serum,^56^ together with the broad tropism, limiting their suitability for in vivo gene delivery. Hence, pseudotyping research is crucial for enhancing retroviral vector infectivity and conferring cell‐specific tropism. Finally, the integration of a viral vector near a proto‐oncogenic or regulatory element primes transformation by affecting gene expression, potentially leading to tumor formation or other diseases. Modifying these vectors to be non‐integrating mitigates insertional mutagenesis,^57^ offering a safer alternative for both in vitro and in vivo gene delivery, especially considering its higher packaging capacity (9 kb) compared with widely used adeno‐associated virus vectors (5 kb).^58^

## 4 DESIGN OF SIN VECTOR

The SIN vector is usually constructed by deleting the enhancer and promoter in the U3 region of the 3′‐LTR, including the CAAT box and TATA box, abolishing the LTR promoter activity.^59^, ^60^, ^61^ After one round of reverse transcription, the deletion is copied to the 5′‐LTR of the proviral DNA, rendering both 5′‐and 3′‐LTRs inactive and thus inactivating transcription of the provirus in the target cell.^51^, ^59^, ^60^, ^61^ Usually, the expression of inserted genes in a SIN vector relies on an internal promoter introduced upstream, often derived from human housekeeping genes, like the human elongation factor 1α (EF1α).^36^, ^61^ The processing of lentiviral vector transcription can be improved by introduction of a post‐transcriptional element derived from the woodchuck hepatitis virus (WPRE), thus resulting an enhanced protein expression.^62^, ^63^, ^64^ Usually, the WPRE is further modified to improve the safety.^65^

**FIGURE 1:**
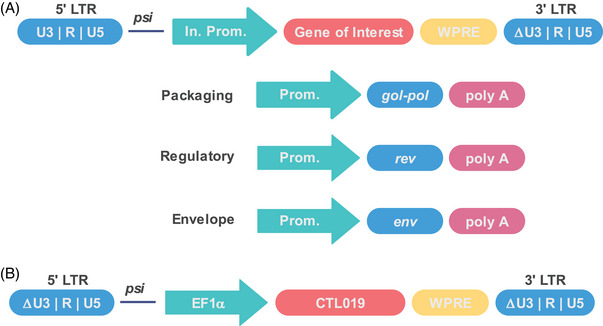
The schematic representation of self‐inactivating (SIN) vectors. (A) The third‐generation lentiviral SIN vector. The third‐generation lentiviral vectors split the genes into four different plasmids, one encoding the gene of interest (GOI), one encoding *gag*, *pol* genes, and another encoding *rev* gene, an additional plasmid encodes the envelope protein. The gamma retroviral SIN vectors contain three plasmids except the regulatory plasmid, because gamma retroviruses are simpler than the lentiviruses and do not need the *rev* gene for the RNA processing. The figure is reproduced with permission.^35^ (B) The transfer vector of the tisagenlecleucel (Kymriah). The transfer vector of tisagenlecleucel deleted U3 region within both 3′‐ and 5′‐LTR. The figure is redesigned according to European Medicines Agency public assessment report of Kymriah.^69^ LTR, long terminal repeat; *psi*, packaging signal; In. Prom., internal promoter; Prom, promoter; WPRE, woodchuck hepatitis virus post‐transcriptional regulatory element.

Proper design of the delivery vectors can remove some potential foreseen risks.^66^ For instance, all the lentiviral vectors used in the approved CAR‐T products belong to the third‐generation SIN vector (Figure 1A), and the splitting of the viral genome into four separate plasmids and deleting the LTR promoter and enhancer greatly reduced the potential of recombination events to occur during vector manufacturing that could generate replication‐competent lentiviruses (RCL). The transfer plasmid contains the GOI and the packaging signal that allows for the GOI to be packaged into the retroviral particles. The generation of RCL is greatly reduced as the *gal, pol, env* genes can only translated into protein.^38^ The LTR contains essential regulatory sequences: the U5 region that contains the polyadenylation signal, followed by the R region that is used as primer during reverse transcription, and the U3 region that contains the viral promoter and enhancer.^36^ The U3 region plays essential role in regulating viral gene expression and in some cases may influence the regulation of neighboring genes.^36^ Thus, deletion of the U3 region could prevent insertional activation or upregulation of host genes, which greatly reduced the oncogene activation.^38^, ^51^, ^67^ However, the deletion also abolishes the transgene expression. To circumvent this problem, internal promoters such as the cytomegalovirus promoter, EF1α promoter, or MND promoter (a synthetic promoter that contains the U3 region of a modified MoMuLV LTR with myeloproliferative sarcoma virus enhancer) are engineered to drive transgene expression.^67^, ^68^ Although deletion of the U3 region of the 3′‐LTR can copy to the 5′‐LTR of the proviral DNA just after one round of reverse transcription, it is noticeable that the transgene vector of the tisagenlecleucel (Kymriah) deleted both the 3′‐and 5′‐U3 region within LTR (Figure 1B). This is an ingenious design as it further reduces the possibility of homologous recombination among different plasmids. Besides, a rational design and careful examination of plasmid backbone lowers homology among vectors, lowering the RCL risk.^66^

## 5 SECONDARY MALIGNANCIES AND RISK MANAGEMENT

On November 28, 2023, United States Food and Drug Administration (US FDA) announced that they have received reports of T‐cell malignancies in patients treated with BCMA‐ or CD19‐directed CAR‐T cells.^70^ They determined that the risk applies to all the six approved CAR‐T products and is investigating the serious outcomes caused by the T‐cell malignancy to evaluate the need for regulatory action.^70^ In January 2024, US FDA asked a black box warning for all these products, highlight the T‐cell malignancies in the prescribing information.^71^ By the end of 2023, the US FDA received 22 reports of T‐cell malignancies linked to CAR‐T treatments.^72^ Among them, three cases that performed sequencing detected CAR gene in the malignant clone,^72^ suggesting a possible connection between the malignancies and retroviral vectors' insertional properties. In 2024, several reports described the rare secondary T‐cell lymphomas following CAR‐T therapy, which can be attributed to multiple reasons, including lentiviral insertion within T‐cell homeostasis genes, the occasional progression of CAR‐T cells themselves to T‐cell lymphoma, and gene mutations related to clonal hematopoiesis.^73^, ^74^, ^75^, ^76^

In China, no T‐cell malignancies have been reported in patients who have infused the CAR‐T products till now. Considering that all the five CAR‐T products approved in China have indicated the possibility of secondary malignancies developed in the “warnings and precautions” part of the package insert upon the marketing approval and also suggested a life‐long monitor for it, the Center for Drug Evaluation (CDE) will not ask for a boxed warning. But still, the CDE suggest that “cases of secondary malignancies (including T‐cell malignancies) after CAR‐T cell therapy have been reported abroad” should be added in the describing part of the secondary malignancies. Also, actions are asked to be taken to strengthen the risk control in clinical trials, and samples such as patients’ tumor tissue or cells, leukocyte apheresis, and CAR‐T products for infusion should be properly retained. Once secondary malignancies occur, tumor tissues or cells should be collected timely and evaluated by various analytical methods such as sequencing to investigate the correlation with the CAR‐T therapy.

In fact, there are already a few reports on the insertional oncogenesis with patients who have received the genetically modified cells generated by retroviral vectors.^76^, ^77^, ^78^, ^79^ There is also report on development of secondary T‐cell malignancies after infusion of CAR‐T cells generated using the *piggy*Bac transposon system.^80^ Therefore, the secondary malignancies might be a potential risk among the integrating vectors, including retroviral vector. There is a report of T‐cell lymphoma developed not due to the CAR insertion, but might be due to the already existing generic mutations of the T cells.^81^ This indicate that the insertion of the CAR gene is not the only cause of secondary malignancies, some other mechanisms also contribute to it. Actually, the risk of oncogenesis depends on many factors, such as the insertional properties of the vectors, the vector design, the vector copy number per cell, the type of transduced cells, the target cell population, and proliferation potential of the target cells. The risk of oncogenesis should be evaluated based on these factors and the integration sites should be determined using representative batch cells, and integration at the critical sites such as near pro‐oncogenes should be avoid.^82^, ^83^ The newly release FDA guidance also gave recommendation regarding the tumorigenicity studies, addressing that the tumorigenicity studies are necessary for pluripotent stem cell–derived products, which have the potential for aberrant cell proliferation, differentiation, and teratoma formation.^84^ For allogeneic cell products, in vitro insertion site analysis before infusion to patients is advisable. Fifteen years follow‐up observation for the insertional oncogenesis caused by retroviral vectors and a life‐long monitor of secondary malignancies are recommended.^70^

The potential generation of RCR also can cause T‐cell malignancies^85^, ^86^; besides, it may cause retroviral diseases such as neurologic disorders and hematologic disorders.^87^ To prevent secondary side effects, suitable vectors can be designed, and RCR testing should be performed on retroviral vectors and their resulting cellular products.

For the vector design, as we discussed above, strategies such as deleting unnecessary accessory genes, separating essential genes into different plasmids, minimizing sequence homology among plasmids, and using SIN‐vector with U3 region deleted at least in the 3′‐LTR can reduce the possibility to form RCR.

In the retroviral vector manufacture process, RCR should be tested with the retroviral vector supernatant and end of production cells (EOPC), and the ex vivo transduced cells.^87^, ^88^ When the vector is produced by a stably transfected producer cell bank system, both cells and supernatant of the master cell bank (MCB) should be tested for RCR.^87^ However, the working cell bank does not require testing if it is derived from a qualified MCB. For the retroviral vectors, both supernatant and EOPC are suggested to be tested, and other testing samples are acceptable if justified. The RCR is not always consistently detected in the same batch of supernatant and EOPC, and the dual testing could assure RCR‐free retroviral vectors.^87^ For supernatant testing, at least 5% of the total supernatant is recommended or it provide enough supernatant that needed to detect 1 RCR/dose with 95% probability. As for cell testing, it is advisable to use 1% or 10^8^ cells (whichever is less) for vector‐producing cells or ex vivo transduced cells.^87^ For the testing assay, the cell testing should be accomplished by co‐culture with a permissive cell line, and the vector supernatant assay should include culture of supernatant on a permissive cell line; both cases allow the amplification of any potential RCR present. Usually, the RCR culture assay include a minimum of five passages, and the RCR assays should be developed to support virus entry, amplification, and particle production specific to the vector design. All assays should include negative and positive controls to evaluate specificity, sensitivity, and reproducibility of the method used. For the vector supernatant, the inhibitory effects should be tested by adding positive control samples to the supernatant. For the ex vivo transduced cells, cell culture or alternative methods like PCR can be used for lot release testing, especially when time is limited. However, to ensure the suitability of the alternative method, it must undergo qualification or validation. The FDA allows reduction or elimination of the RCR testing upon ex vivo transduced cells in the case that the sponsor provide enough supporting information, including the vector design, vector testing according to current guidance, the accumulated manufacturing, and clinical experience of consistently RCR‐free cell products.^87^

For patient monitoring, US FDA recommends two RCR detection methods: serologic detection of the RCR‐specific antibodies, such as the antibodies to retroviral *gag*‐encoded protein P30,^89^, ^90^ or qPCR analysis of RCR‐specific DNA sequences like the viral envelope sequence.^91^, ^92^ There are some challenges toward these two detection methods. The qPCR method might give false‐negative result if the RCR sequence mutates, while the serology method might fail to develop antibodies or develop antibodies to vector protein infused during treatment.^93^, ^94^ Sponsors can choose assay according to the vector type, mode of vector administration, and the clinical indication.^87^ To identify delayed adverse events and mitigate long‐term risks, a 15‐year follow‐up is advised.^82^ Patient samples should be analyzed prior to treatment and then at 3, 6, and 12 months after treatment and annually for up to 15 years.

Due to limited follow‐up data and product understanding, some potential risks may be underestimated. Collecting more data to characterize and monitor risks is crucial. Low‐risk rates can be further reduced with technological advances and delivery strategies. New emerging testing strategies and methods will also aid in understanding mechanisms and potential risks, enabling better risk management. Also, instructions should be given to assure a proper collection and storage of the patients’ samples in order to generate useful data to analyze and evaluate the risk.

The risk of oncogenesis is an issue accompanied with the genetically modified cells. For patients who have serious life‐threatening malignancies or diseases with limited survival times, the benefits of CAR‐T therapy clearly surpass the risks of secondary malignancies.^95^, ^96^ Additionally, follow‐up studies indicate that the incidence of T‐cell malignancies with CAR‐T therapy is much lower compared with some other treatments.^97^ Therefore, it is widely believed that in most scenarios, the advantages of CAR‐T therapy exceed its potential risks.

There are also other potential risks caused by using retroviral vector‐based CAR‐T therapy, such as reactivation of the human latent viruses^98^, ^99^ and the CAR gene integrated into the B cells.^100^ However, with a deeper understanding of these risks, proper monitoring, and technological advancements, we believe safety can be enhanced, benefiting both patients and the industry.

## 6 CONCLUSION AND FUTURE PERSPECTIVE

CAR‐T therapy has revolutionized hematologic cancer treatment, offering hope for patients with life‐threatening malignancies. Also, notable efforts are underway to extend this success to non‐malignancy conditions such as autoimmunity, metabolic disease, fibrosis, and senescence.^101^ The therapy's application is broadening, requiring improvements in safety, efficiency, and quality of cell products. Modifications toward site‐specific integration, cell specific tropism, non‐integrating vector, high transduction efficiency, and construction of stable producing cell lines for SIN vectors will improve the safety and allow for the safe broadening of clinical applications. Alternative delivery methods like stable‐integrating non‐virus vectors, CRISPR/Cas9, and non‐integrating viral vectors or mRNA can enhance safety and broaden applications. Technological advancements and risk management will enhance safety and broaden applications of genetically modified cell products.

## AUTHOR CONTRIBUTIONS

Huifang Yin wrote the manuscript, Xuejing Wei revised, and Huifang Yin finalized the manuscript. All authors contributed to the discussions and have read and approved the article.

## CONFLICT OF INTEREST STATEMENT

The authors declare no conflicts of interest.

## Data Availability

Not Applicable.
